# Ago-2-Mediated Slicer Activity Is Essential for Anti-Flaviviral Efficacy of RNAi

**DOI:** 10.1371/journal.pone.0027551

**Published:** 2011-11-10

**Authors:** Shuiping Chen, Harendra S. Chahar, Sojan Abraham, Haoquan Wu, Theodore C. Pierson, Xiaozhong A. Wang, N. Manjunath

**Affiliations:** 1 Department of Biomedical Sciences, Center of Excellence in Infectious Disease Research, Paul L. Foster School of Medicine, Texas Tech University Health Sciences Center, El Paso, Texas, United States of America; 2 Viral Pathogenesis Section, Laboratory of Viral Diseases, National Institute of Allergy and Infectious Diseases, National Institutes of Health, Bethesda, Maryland, United States of America; 3 Department of Biochemistry, Molecular Biology and Cell Biology, Northwestern University, Evanston, Illinois, United States of America; National Institute of Health, United States of America

## Abstract

RNA interference can be mediated by fully complementary siRNA or partially complementary miRNA. siRNAs are widely used to suppress viral replication and the fully complementary siRNA bound Ago-2 in the RISC is known to degrade the target RNA. Although other argonaute proteins lacking slicer activity can also bind oligonucleotides with both si and miRNA structures, whether they can also contribute to antiviral effects is not entirely clear. We tested si and miRNA structured oligos for target repression in dual luciferase assays as well as for inhibition of Dengue and West Nile virus replication in ES cells expressing individual Ago proteins. In luciferase assays, both fully complementary and partially complementary oligos effectively repressed their targets in all individual Ago expressing cell lines, although the efficacy with fully complementary oligos was higher in Ago-2+ cells. However, partially complementary oligos had no effect on virus replication in any cell line, while fully complementary siRNAs were highly effective in Ago-2 expressing, but not in cells expressing other Ago proteins. This occurred irrespective of whether the target sequences were located in the coding region or 3′UTR of the virus. We conclude that Ago-2 slicer activity is essential for anti-viral efficacy of siRNAs and miRNA-mediated translational repression/transcript destabilization is too weak to suppress the abundantly expressed flaviviral proteins.

## Introduction

RNA interference (RNAi) is a phenomenon where small double stranded RNAs mediate sequence-specific regulation of gene expression. Essentially, RNAi can be induced either by endogenously encoded small RNAs called microRNAs (miRNAs), endogenously generated small interfering siRNAs (siRNA) or exogenously introduced siRNAs [1], [2], [3], [4] **.** In either case, the 21–23 nucleotide dsRNAs associate in the cytoplasm with the RNA-induced silencing complex (RISC), whereupon one of the two RNA strands (passenger strand) is discarded and the other guide strand guides the RISC to mediate sequence-specific degradation of the corresponding mRNA (in the case of siRNAs) and/or translational repression/transcript destabilization by binding to the 3′ untranslated region (UTR) (in the case of miRNAs) [5], [6].

While siRNAs are designed to be fully complementary to the coding region of the mRNA target to be silenced, miRNAs are imperfectly complementary, generally having sequence matches in the 5′ 2-8nt seed sequence to the 3′UTR of the target mRNA. Also, while siRNAs primarily degrade the target mRNA, miRNAs lead to translational repression/mRNA destabilization (reviewed in [7]). Thus, although both mi and siRNAs use the same RNAi machinery, the exact mechanism of gene silencing is different. Argounate (Ago) proteins are key constituents of the RISC and mammalian cells contain 4 Ago proteins, Ago1-4. Recent studies suggest that both si and miRNAs are indiscriminately loaded into all 4 Ago proteins in mammalian cells [8], [9]. However, since only mammalian Ago-2 has slicer activity, the role of other Ago proteins in the siRNA pathway is unclear.

One of the important applications of siRNAs is to suppress viral infection and proof of concept studies in cell lines, and various animal models including mice, monkeys and chimpanzees suggest that virtually any viral infection can be effectively silenced (reviewed in [10]). In fact, the recently concluded Phase II human clinical trial for RSV infection also underscores the antiviral potential of siRNAs [11]. Several cellular miRNAs have also been reported to affect the replication of different (primate foamy virus, influenza virus, hepatitis B, HIV and HSV) viruses [12], [13], [14], [15], [16], [17], [18]. Generally the miRNAs have perfect or imperfect homology in the seed region to their target sites in the viral coding region or 3′UTR. Despite this, the miRNAs repress the virus by either transcript degradation (even with only seed matches) or translational repression. Thus, the exact mechanism of miRNA mediated viral repression and which Ago proteins are involved remain poorly understood. As described earlier, siRNAs are designed to be perfectly homologous to the target sequence and the targets are therefore amenable to be degraded by Ago-2. However, whether siRNAs bound to other Ago proteins or synthetic siRNAs designed to mimick miRNA structure (being only partially complementary at the “seed sequence” in the target) can also be used to suppress viral infection is not known [19], [20]. Although miRNAs were thought to bind to their target in the 3′UTR, recent data from several laboratories suggest that seed matches in the coding region can also serve as effective targets for miRNAs [21], [22], [23], [24]. In fact as mentioned earlier, several cellular miRNAs that are known to repress viral replication also bind to viral coding regions. If miRNA-mediated translational repression/transcript destabilization can be used in conjunction with siRNA mediated transcript degradation, it could enormously enhance RNAi antiviral efficacy. Thus, in this study, ES cells expressing individual Ago proteins were tested for suppression of viral replication by fully complementary siRNAs or partially complementary oligos resembling miRNAs.

## Results

### All the 4 mammalian Ago proteins can mediate target repression by oligonucleotides with both si and miRNA structures in reporter assays

**Figure 1:**
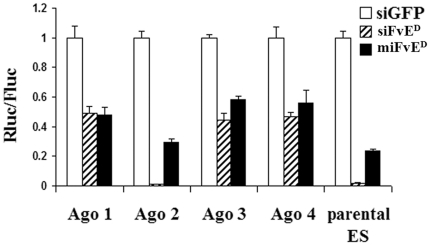
All 4 Ago proteins bind both fully complementary siRNA and partially complementary miRNA resembling oligos and repress target gene in reporter assays. Parental ES cells expressing all ago proteins and ES cells expressing individual Ago proteins were transfected with dual luciferase reporter vector containing dengue virus siRNA target sequences in the Renilla luciferase 3′UTR along with fully or partially complementary dengue virus siRNAs and the dual luciferase expression measured after 24 h of transfection. Rluc/Fluc expression normalized to control irrelevant GFP siRNA is shown. Error bars represent mean of quadruplicates +/− SD.

We have previously identified siRNAs that potently suppress dengue and West Nile virus infection in vitro and in vivo [25], [26], [27]. To test whether the different Ago proteins can bind si and miRNA structured oligonucleotides and mediate target repression, we used a dengue virus siRNA as base. We constructed a dual luciferase reporter with 1 copy of fully complementary target sites for dengue siRNA (siFvE^D^) in the renilla luciferase 3′UTR. This plasmid was transfected along with fully complementary or partially complementary dengue virus siRNA (sequence differing at nts 9-11, Table 1) into ES cells expressing only Ago-1, Ago-2, Ago-3 or Ago-4 [9] and analyzed for target repression after 24h. Partially complementary dengue miRNA mimicking siRNA could repress the target (40–60%) in cells expressing individual or all the Ago proteins (Fig. 1). Although fully complementary siRNA also repressed the target to a similar extent in Ago-1, Ago-3 and Ago-4 expressing cells, the target repression was more profound in Ago-2 and in all 4 Ago expressing cells (>90%). Thus, oligonucleotides with both si and miRNA structure can bind to all the Ago proteins and repress targets, although the efficacy is highest for siRNAs in Ago-2 expressing cells.

**Table 1:** Sequence of siRNA and miRNA mimicking oligonucleotides.

siRNA/miRNA	sense strand sequence (5′→3′)
siFvE^D^	ggatgtggattatttggaa
miFvE^D^	ggatgtgg***taa***atttggaa
siWN-3UTR	tgtagtgttcatagcaatt
miWN-3UTR	tgtagtgt***agt***tagcaatt

SiRNAs were designed to completely match the target sequence, while miRNA mimicking siRNAs were designed to contain central 3 mismatches (nts 9-11-nts depicted in bold letters).

### Only Ago-2 bound fully complementary siRNAs suppress viral replication

**Figure 2:**
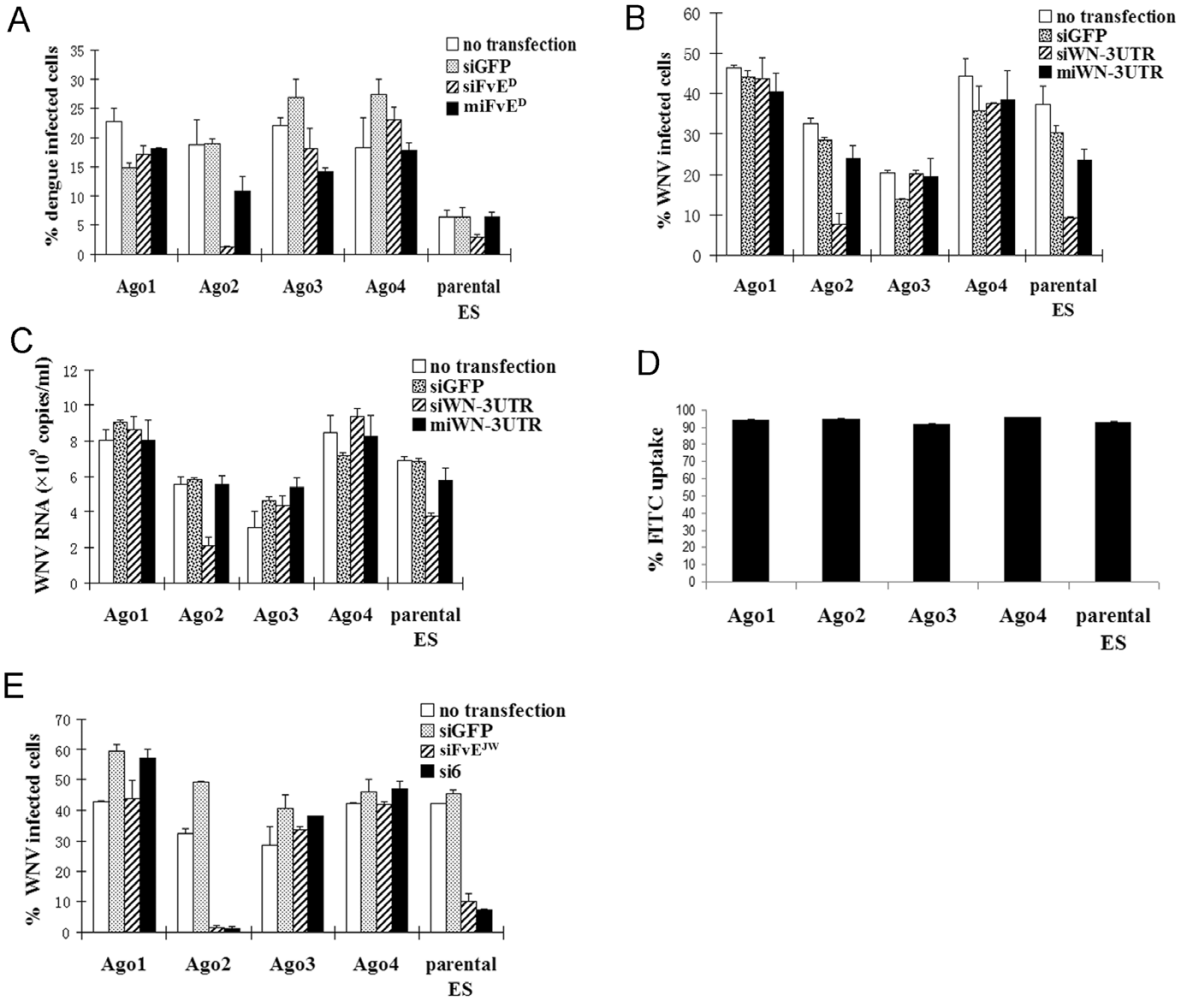
Only Ago-2 bound fully complementary siRNA shows antiviral activity. A, B) ES cells expressing individual Ago proteins were transfected with fully or partially complementary siRNAs, infected with dengue virus (A) or WNV (B) and virus replication measured by FACS analysis after 72 h following infection. siGFP was used as negative control. C) WNV particles released into ES supernatant in (B) were quantified by QRT-PCR. D) Transfection efficacy in different ES cells was assessed using FITC labeled siRNA. E) ES cells were transfected with 2 different anti-WNV siRNAs, infected with WNV and virus replication determined as in A). Error bars represent mean of duplicates +/- SD.

Because in the reporter assay, both si and miRNA structured oligos repressed targets in all individual Ago expressing cells, we tested if these oligos could also suppress viral infection. First we determined that all individual Ago expressing cells were susceptible to virus infection, although for unknown reason, the parental ES cells expressing all Ago proteins showed the lowest levels of infection (Fig 2A). To test antiviral efficacy of RNAi, all or individual Ago expressing cells were transfected with dengue si or mi RNA mimicking oligos, infected with dengue virus 24h later and assessed for infection 72h after infection. To detect infection, cells were stained with a dengue virus envelope-specific monoclonal antibody and analyzed by FACS. As shown in Fig 2A, the fully complementary siRNA effectively suppressed the virus replication in Ago-2 and all Ago protein expressing parental cell line. However, the siRNA did not significantly affect virus replication in Ago-1, Ago-3 or Ago-4 expressing cells and the miRNA structured oligos failed to significantly suppress virus replication in any of the individual Ago expressing cell line.

One reason why the miRNA resembling siRNA failed to suppress virus replication in the above experiment is that both the si and miRNAs were designed to target the coding region in the virus (encoding the viral envelop gene), whereas endogenous miRNAs generally target the 3′UTR sequences. To test if this is indeed the case, we also tested si/miRNA resembling oligos that target the viral 3′ UTR. Initially we tested several potential siRNAs targeting West Nile virus 3′ UTR and identified one that potently suppressed viral replication in HeLa cells (not shown). We used this siRNA and its counterpart miRNA structure (Table 1) for virus inhibition in individual Ago expressing cells by FACS analysis as well as testing the culture supernatants for released virus particles by qPCR. Even in this case, the completely complementary siRNA effectively suppressed virus replication in all 4 Ago expressing parental cell line and Ago-2 only expressing cells, but failed to significantly inhibit the virus in Ago 1, Ago 3 and Ago 4 expressing cells (Fig 2B and 2C). Moreover, the miRNA resembling siRNA failed to suppress virus replication in all cell lines. These results are not due to variability in the transfection efficiency in different cell lines, because all the individual Ago expressing cells were equally amenable for transfection with FITC labeled siRNA (Fig. 2D). Taken together, our results suggest that siRNAs designed to mimic miRNA structure fail to inhibit flavivirus replication whether the target is in the coding region or 3′ UTR.

We also tested 2 additional siRNAs that we had earlier identified as potent suppressors of West Nile virus (WNV) [26], [28]. Even in this setting, both the tested siRNAs effectively inhibited WNV infection in Ago-2 expressing cells and the parental cell line, while they were ineffective in other Ago expressing cells (Fig. 2E). Thus Ago-2 mediated target cleavage appears to be essential for antiviral efficacy of siRNAs. These results also suggest that siRNAs bound to Agos 1, 3 and 4 only serve to dilute the siRNA effect.

### Although P-bodies are lost following flaviviral infection, RNAi function is not affected

**Figure 3:**
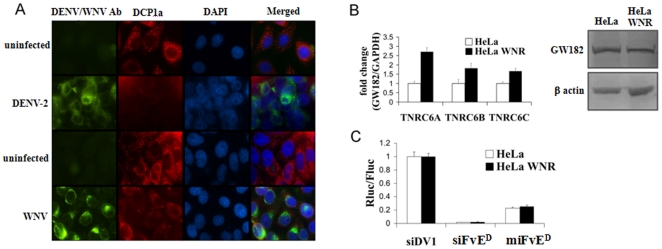
Flavivirally infected cells lose microscopic P bodies, but not GW182 and exhibit both si and miRNA-mediated target repression. A) Uninfected and dengue or West Nile virus infected HeLa cells were stained with DAPI, DCP1a and dengue/WNV antibody and examined for P bodies by microscopy. B) Control HeLa cells and HeLa cells expressing WNV replicon (HeLa WNR) were tested for GW182 isoform expression at mRNA level by QRT-PCR (left) and protein level by Western blot (right). C) West Nile replicon expressing HeLa cells were transfected with luciferase reporter with si/miRNA and analyzed as in Fig 1a. siDV1 represents control irrelevant siRNA. Error bars represent mean of quadruplicates +/− SD.

Many of the proteins involved in the RNAi pathway accumulate in discrete cytoplasmic bodies called the processing or P bodies [29]. Moreover GW182, a key component of the P- body, associates with all Ago proteins as well as with mRNA and is essential for miRNA-mediated target repression [30]. A recent report suggests that flavivirus infection leads to loss of stress granules as well as P bodies [31]. We therefore tested if P bodies are indeed lost following flaviviral infection and if this could be why the miRNA mimicking siRNAs are ineffective in silencing viral replication. Compared to control HeLa cells, in HeLa cells infected with dengue or WNV, P bodies were dramatically diminished as revealed by staining with DCP-1 antibody (Fig 3A). Similar results were also seen after transfection of HeLa cells with WNV replicon (not shown), confirming the previous report as well as showing that introduction of the replicon is sufficient to induce the loss of P bodies. To test if the loss of microscopically visible P bodies is due the loss of GW182, we also performed qRT-PCR and Western blot for GW182. GW182 mRNA as well as protein levels were not decreased in replicon containing cells as compared to control HeLa cells (Fig 3B). We also tested if RNAi machinery is ineffective because of loss of P bodies. si/miRNA mimicking oligonucleotides were transfected into dengue replicon expressing cells along with the luciferase construct containing the target sequence in the 3′UTR. As shown in Fig 3C, both si and miRNA structured oligos were effective in repressing luciferase although as expected, siRNA was more effective than miRNA structured oligo. Collectively, these results suggest that although microscopic P bodies were diminished in infected cells, the GW182 protein itself was not and it did not affect the RNAi response. This finding is also consistent with a recent report in drosophila S2 cells that suggests that P-body formation is a consequence rather than the cause of RNAi-mediated silencing [32].

## Discussion

Our results suggest that although effective in reporter assays, completely complementary siRNAs loaded to Agos1, 3 and 4 and siRNAs mimicking miRNAs with only seed matches to the viral targets in the coding region or 3′UTR loaded to any of the Agos fail to suppress an acute flaviviral infection.

Because only Ago-2 has slicer activity and also because extensive pairing with the target is required for slicing, siRNAs bound to Ago-2 can degrade the target mRNA whereas siRNAs bound to Agos 1, 3 and 4 as well as miRNAs bound to any Ago protein can only mediate target repression by translational repression/transcript destabilization [5]. Intrinsically, miRNAs are meant to fine tune gene expression and their activity reduces the target proteins only to modest levels. Moreover, after degrading a target, the Ago-2 bound siRNA is free to attack the next target molecule, whereas oligos bound to other Agos presumably need to interact continuously to mediate translational repression/destabilization. Thus, Ago-2 mediated siRNA effects are likely to be catalytic and thus more robust than siRNAs bound to other Agos as well as the miRNA effects. Our results are entirely in agreement with this hypothesis. Our results have a number of implications: since siRNAs are distributed between all Agos in mammalian cells of which only Ago-2 bound siRNA has antiviral effect, most of the input siRNAs are ineffective and are wasted, although the actual portion bound to each Ago when all Agos are expressed is not known. In other words, siRNAs required for actual antiviral effect is likely to be only a fraction of what appears to be needed. Moreover, the siRNAs bound to Agos 1, 3 and 4 may interfere with the binding of the endogenous miRNAs, thereby resulting in toxicities. Thus, if siRNAs can be designed to be only loaded to Ago-2, it might both improve antiviral efficacy and at the same time reduce toxicities. In this regard, cellular pre-miRNA 451 has recently been shown to bypass dicer cleavage to be directly processed by Ago-2 [33]. Conceivably, antiviral siRNAs could be designed in the context of pre-miRNA 451 that is directly processed by Ago2 and incorporated into Ago-2 bound RISC.

Many viruses also encode miRNAs that are completely or partially homologous with their viral targets and cellular miRNAs have also been proposed to affect viral replication [19], [20], [34]. Although in several cases, the targeted region appears to be in the 3′ UTR, several cellular miRNAs are known to target the coding region to repress the target gene [21], [22], [23], [24]. Although several cellular miRNAs have also been reported to affect many viral replication by binding to viral coding region or 3′UTR, our results suggest that synthetic siRNAs designed to mimic miRNA having a seed match to the viral sequences may not be enough for antiviral activity in an acute flaviviral infection. This may be because the miRNA-like effects are generally mild and only serve to moderately reduce the target protein levels, which may not be able to affect the abundantly expressed viral proteins. Recently, dual functional siRNAs that are both completely complementary to a viral target and at the same time possess seed matches in the 3′UTR have been proposed to improve the functionality of siRNA [35]. Again, the additive effect of seed matches in the coding region is likely to be minimal in suppressing flaviviral infection.

Many of the proteins involved in the RNAi pathway accumulate in P bodies [29]. In HIV infection, P bodies are used to suppress infection: miRNA 29 family that represses the viral replication as well as the viral transcripts associate with P bodies and disruption of P body components by siRNA results in enhancing HIV replication [36], [37]. In contrast, Dengue and West Nile virus infection result in the progressive loss of P bodies and resistance to stress granule formation. Although inhibition of stress granule formation may be due to the recruitment and sequestration of TIA-1 and TIAR into the viral replication complex [31], the reason for loss of P bodies is not known [31]. Our results suggest that disappearance of microscopic P bodies is not due to the loss of GW182 protein in infected cells. It is possible that similar to TIAR, GW182 could also be recruited into the replication complex. Alternatively, other essential proteins needed for P body formation such as LSm proteins, RCK/p54 or Ge-1 [29] may be affected. Whatever the mechanism of loss of P bodies, our results suggest that GW182 protein, which is essential for miRNA-mediated repression is not affected and both the siRNA and miRNA pathways remain intact after flaviviral infection. Thus, lack of antiviral effect of miRNA-structured siRNAs is not due to loss of P bodies, but most likely, as mentioned earlier because the miRNA mimicking siRNA effects are too weak to suppress the abundantly expressed flaviviral proteins.

In summary, robust suppression of viral infection can only be achieved by completely complementary siRNA bound Ago-2-mediated target cleavage and miRNA mediated translational repression/mRNA destabilization or other Ago proteins appear to have no role in the anti-flaviviral RNAi.

## Materials and Methods

### siRNAs and miRNA mimicking oligonucleotides

SiRNA and miRNA mimicking oligos were obtained from Dharmacon (Lafayette, CO). The sense strand sequences from 5′ to 3′ end were: siFvE^D^ (siRNA against dengue virus) ggatgtggattatttggaa; miFvE^D^ (miRNA mimic) ggatgtggtaaatttggaa; siWN-3UTR (siRNA against WNV 3″ UTR) tgtagtgttcatagcaatt; miWN-3UTR (miRNA mimic against WN 3′UTR) tgtagtgtagttagcaatt; siFvE^JW^ (siRNA against WNV) gggagcattgacacatgtgca; si6 (siRNA against WNV), ctgtgacattggagagtca; siDV1 (negative control siRNA) cagcatattgacgctggga; siGFP (negative control siRNA) ggctacgtccaggagcgca.

### Dual-luciferase constructs and assay

Reporter was constructed by cloning the DNA oligos containing the target sequence between the XhoI and NotI sites downstream of the Renilla luciferase gene in the psiCHECK2 vector (Promega, Maddison, WI). The DNA oligos for the dengue siRNA (siFvE^D^) target ggatgtggattatttggaa was synthesized at IDT.

Ago1-4 expressing ES cells and HeLa cells were cultured as described previously [9]. One day before transfection, the cells were trypsinized and diluted to 10^5^ cells/ml and seeded in 96 well plates in a volume of 100 µL/well. Reporter plasmids (20 ng of psiCHECK2 plasmid harboring the target region) together with siRNA or miRNA structured oligos (1 pmol siRNA/miRNA oligos) were cotransfected using Lipofectamine 2000 (Life Technologies, Carlsbad, CA). After 24 h, Renilla and firefly luciferase activities were measured according to the manufacturer's instructions using the Dual-Glo luciferase assay kit. The ratio of Renilla to firefly luminescence was normalized to the negative control siRNA (siGFP or siDV1). Luminescence activity values represented an average of four replicates.

### Testing siRNA/miRNAs for antiviral activity

The antiviral activity of si/miRNAs was tested as previously described [25], [26]. Briefly, ES cells were seeded in 48-well plates at 4×10^4^ cells per well one day before transfection. The siRNAs (8 pmol) were transfected into cells with RNAiMax (Invitrogen) per the manufacturer's instructions. 24 hours after transfection, the cells were infected with Dengue-2 (NGC strain, ATCC) or West Nile virus (B956 strain, ATCC) (moi = 1-5). 72 hours later, the cells were stained with anti-flavivirus envelope specific antibody (4G2, ATCC), followed by flow cytometric analysis to determine inhibition of virus replication.

### Hela cells stably expressing WNV replicon

WNV replicon pWIIREPG-Z [38] was transfected into HeLa cells using lipofectamine 2000. The stably transfected HeLa cells were selected using DMEM medium supplemented with 300 μg/ml Zeocin for 5–7 days followed limited dilution cloning. One clone (HeLa-WNR) expressing GFP and harboring full-length WNV replicon (as assessed by RT-PCR) was selected.

### Immunostaining and Western blot

Untransfected, WNV replicon transfected and WNV or Dengue virus infected HeLa cells were immunostained for P bodies. Cells grown on cover slips were washed in PBS, fixed in 4% paraformaldehyde, permeabilized with 0.5% Triton X-100, and blocked (2% bovine serum albumin, 5% normal horse serum, and 10 mM glycine in phosphatebuffered saline). The cells were then incubated with a rabbit anti -DCP1a antibody (Abcam) and West Nile or DENV-2 specific antibodies (US Biologicals), followed by incubation with appropriate secondary antibodies (AlexaFluor 594-conjugated anti-rabbit antibody and fluorescein isothiocyanate-conjugated anti-mouse antibody (Invitrogen). The cells were visualized for WN/DENV-2 and DCP1a staining using a Nikon Eclipse fluorescence microscope.

For Western blot analysis, normal, replicon expressing and virus infected cell lysates were electrophoresed on 12% Nu-PAGE gels (Invitrogen) and electroblotted onto polyvinylidene difluoride membranes (Immobilon-P transfer membrane; Millipore). Following a blocking step with Tris-buffered saline containing 0.1% Tween-20 and 5% dry milk, the membranes were incubated with a GW182 (TNRC6A) antibody (Abcam), followed by horseradish peroxidase-conjugated secondary antibody (Peirce). Bound horseradish peroxidase was visualized with an ECL substrate kit (Thermo Scientific). Membranes were stripped and reprobed with a rabbit anti β-actin antibody (Cell Signaling) as a loading control.

### QRT-PCR

Total RNA was isolated from HeLa cells and stable HeLa expressing WNV replicon or from WNV infected ES cell supernatants using RNeasy kit (Qiagen). Reverse transcription was performed using superscript III first-strand synthesis superMix (Invitrogen). Quantitative real-time PCR was performed using a SYBR green PCR master Mix (Applied Biosystems) on 7900HT fast real-time PCR system (applied Biosystems). Amplification conditions were as follows: 95°C for 10 min, followed by 40 cycles of 95°C for 15 s, and 60°C for 1 min. The forward and reverse primers to amplify GAPDH were atggggaaggtgaaggtcg and gggtcattgatggcaacaatatc, and that for TNRC6 A, B and C isoforms were gaaatgctctggtccgctaca & atctcctcttcactggcaaactca; ggagcaaaagcacaccacctg & tgctcctgtatcatccatctcg; cccgccgcacctgtctct & ctgctgctctttggtctgc, respectively and for WNV were atcgccggacttatgttcg & ctttcgctagagcctgtgattt. Relative TNRC6 mRNA expression was normalized with GAPDH mRNA and calculated using the δCt method.

### Statistical analysis

Student's t test (two-tailed, assuming equal variances on all experimental data sets) was used to compare two groups of independent samples.
